# Supplementary material for: Subunit promotion energies for channel opening in heterotetrameric olfactory CNG channels
Source: PLoS Comput Biol. 2022 Aug 23;18(8):e1010376. doi: 10.1371/journal.pcbi.1010376 (PMC9512249; doi:10.1371/journal.pcbi.1010376)
Supplement: S1 Fig — (DOCX) [file pcbi.1010376.s001.docx]

**
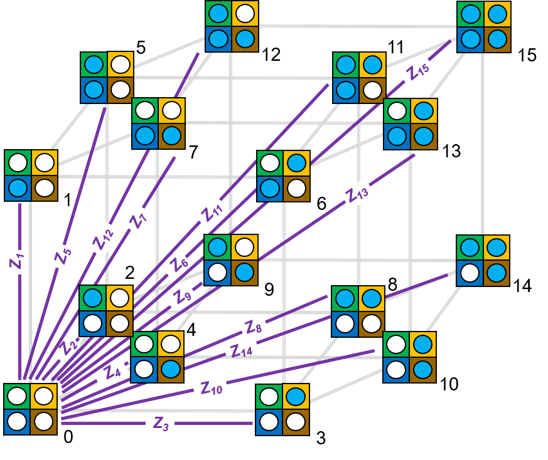
**

**Fig. S1. Virtual equilibrium association constants in the 4D hypercube.** The hypercube corresponds to that in Fig. 1E. The 15 violet lines from state C0 to each corner indicate the 15 virtual equilibrium association constants *Z_1_*-*Z_15_*, specifying the only independent parameters. *Z_1_*-*Z_15_* were used to compute the 32 *K_x_* for wt channels by respective ratios (Tables S2, S4, S5).
