# Supplementary material for: Subunit promotion energies for channel opening in heterotetrameric olfactory CNG channels
Source: PLoS Comput Biol. 2022 Aug 23;18(8):e1010376. doi: 10.1371/journal.pcbi.1010376 (PMC9512249; doi:10.1371/journal.pcbi.1010376)
Supplement: S2 Fig — (DOCX) [file pcbi.1010376.s002.docx]

**
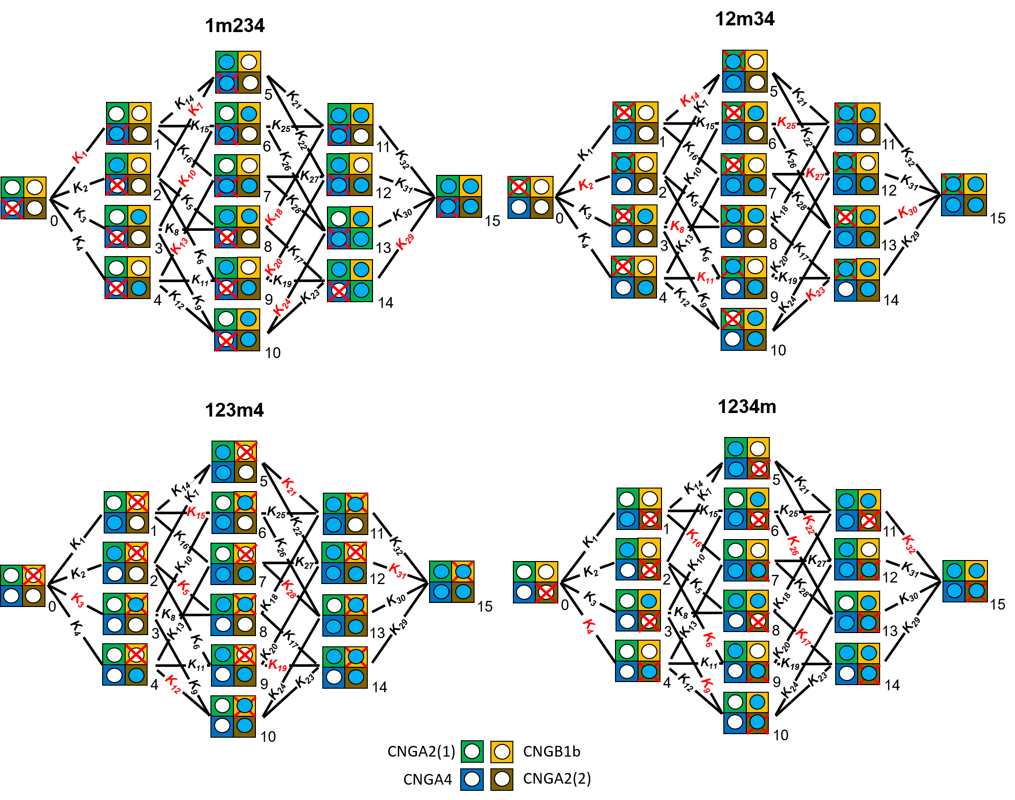
**

**Fig. S2.** **HA models for four concatamers containing one disabled binding site.** The concatamers build together with the model in Figure 1D and the models in Figures S3 to S5 the 16 models used for the global fit. Blue circles represent a ligand, white circles an empty binding site and a red cross on a white circle a disabled binding site. Equilibrium association constants for ligand binding, *K_x_*, are indicated in black for a wt and red for a disabled subunit. One HA model contains 32 *K_x_*, either black or red. Shown is only the network for ligand binding. Each kinetic model has to be complemented by the scheme of the closed-open isomerizations shown at the bottom of Figure 1D.
