# Supplementary material for: Subunit promotion energies for channel opening in heterotetrameric olfactory CNG channels
Source: PLoS Comput Biol. 2022 Aug 23;18(8):e1010376. doi: 10.1371/journal.pcbi.1010376 (PMC9512249; doi:10.1371/journal.pcbi.1010376)
Supplement: S4 Fig — (DOCX) [file pcbi.1010376.s004.docx]

**
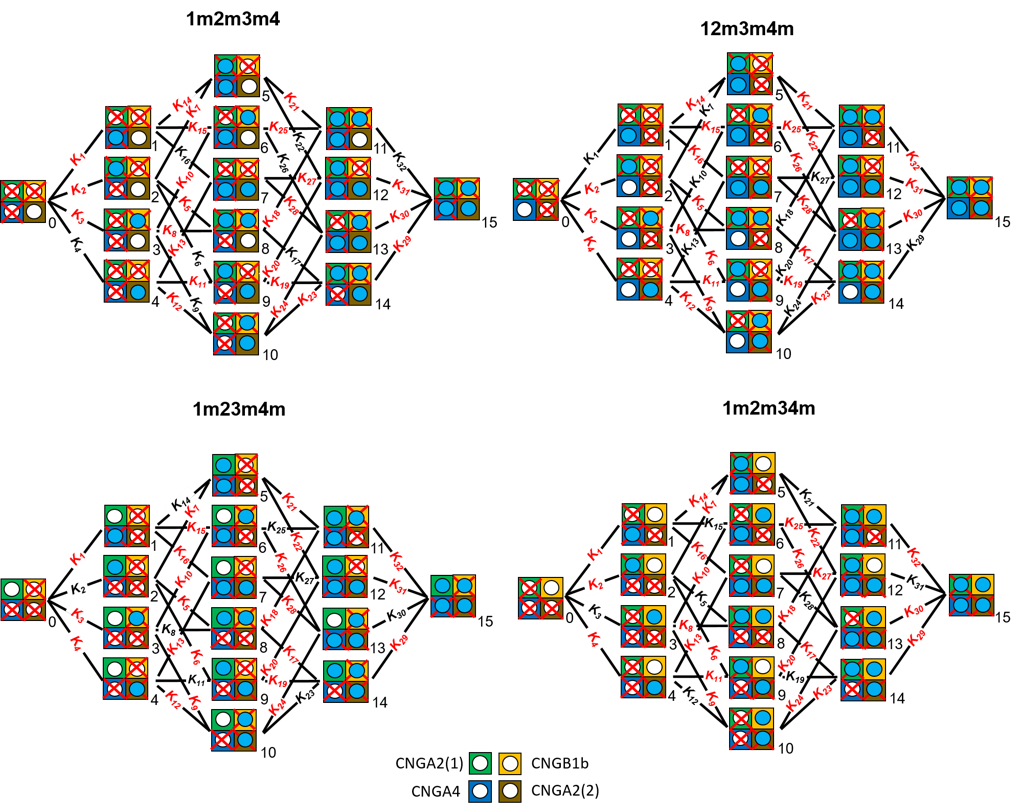
**

**Fig. S4. HA models for four concatamers containing three disabled binding sites.** The concatamers build together with the model in Figure 1D and the models in Figures S2, S3, and S5 the 16 models used for the global fit. For further explanation see legend to Figure S2.
