# Supplementary material for: Subunit promotion energies for channel opening in heterotetrameric olfactory CNG channels
Source: PLoS Comput Biol. 2022 Aug 23;18(8):e1010376. doi: 10.1371/journal.pcbi.1010376 (PMC9512249; doi:10.1371/journal.pcbi.1010376)
Supplement: S5 Fig — (DOCX) [file pcbi.1010376.s005.docx]

**
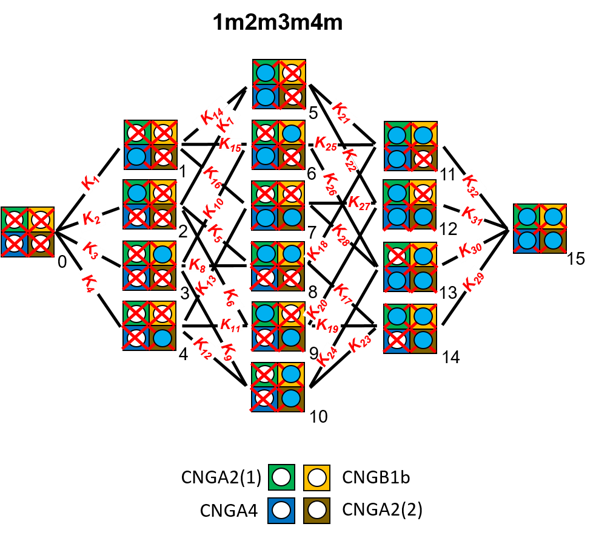
**

**Fig. S5. HA model for the concatamer containing four disabled binding sites.** The concatamer builds together with the model in Figure 1D and the models in Figures S2 to S4 the 16 models used for the global fit. For further explanation see legend to Figure S2.
