# Supplementary material for: Subunit promotion energies for channel opening in heterotetrameric olfactory CNG channels
Source: PLoS Comput Biol. 2022 Aug 23;18(8):e1010376. doi: 10.1371/journal.pcbi.1010376 (PMC9512249; doi:10.1371/journal.pcbi.1010376)
Supplement: S6 Fig — (DOCX) [file pcbi.1010376.s006.docx]

**
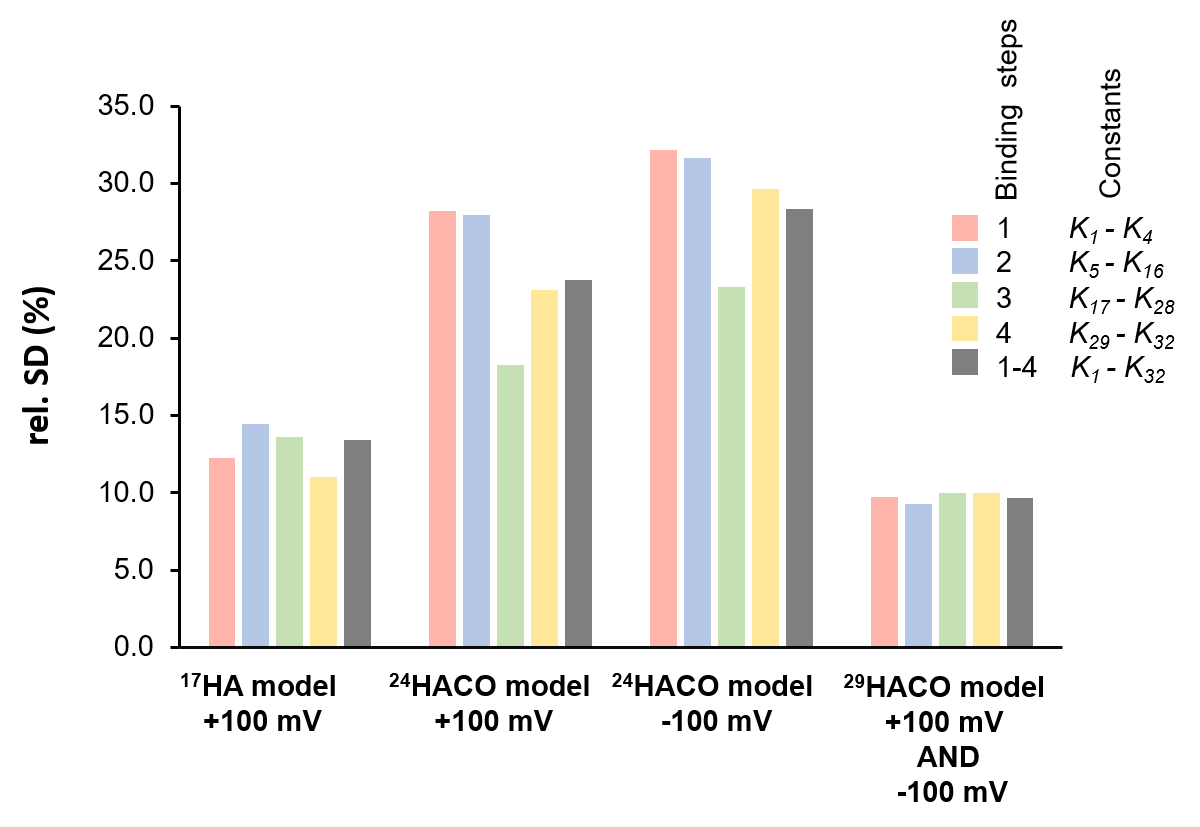
**

**Fig. S6. Comparison of errors obtained by fits with different models.** For the fits with the ^17^HA model [1], ^24^HACO model at +100 mV, ^24^HACO model at -100 mV, and ^29^HACO model at +100 mV AND -100 mV, the errors are plotted as percentage for the averaged first, second, third and fourth binding steps as well as for all 32 binding constants.

**References**

1. Schirmeyer J, Hummert S, Eick T, Schulz E, Schwabe T, Ehrlich G, et al. Thermodynamic profile of mutual subunit control in a heteromeric receptor. Proc Natl Acad Sci U S A. 2021;118(30). Epub 2021/07/25. doi: 10.1073/pnas.2100469118. PubMed PMID: 34301910; PubMed Central PMCID: PMCPMC8325370.
