# Supplementary material for: Subunit promotion energies for channel opening in heterotetrameric olfactory CNG channels
Source: PLoS Comput Biol. 2022 Aug 23;18(8):e1010376. doi: 10.1371/journal.pcbi.1010376 (PMC9512249; doi:10.1371/journal.pcbi.1010376)
Supplement: S7 Fig — (DOCX) [file pcbi.1010376.s007.docx]

**
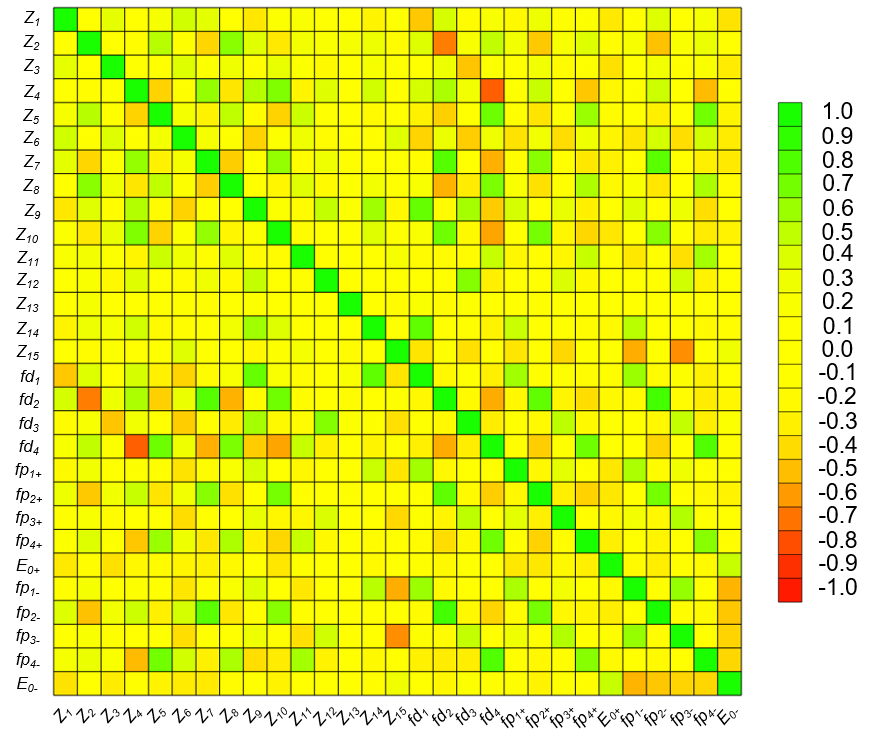
**

**Fig. S7. Color-coded matrix of correlation coefficients for the fit with the ^29^HACO model.** The 29 parameters are *Z_x_, fd_1_-fd_4_, E_0+_*, *fp*_1+_-*fp*_4+_, *E_0-_*, and *fp*_1-_-*fp*_4-_*.* The main diagonal illustrates the correlations of the parameters with themselves, resulting in correlation coefficients of 1. With rare exceptions most correlation coefficients are between -0.3 and 0.3.
