# Supplementary material for: Subunit promotion energies for channel opening in heterotetrameric olfactory CNG channels
Source: PLoS Comput Biol. 2022 Aug 23;18(8):e1010376. doi: 10.1371/journal.pcbi.1010376 (PMC9512249; doi:10.1371/journal.pcbi.1010376)
Supplement: S8 Fig — (DOCX) [file pcbi.1010376.s008.docx]

**
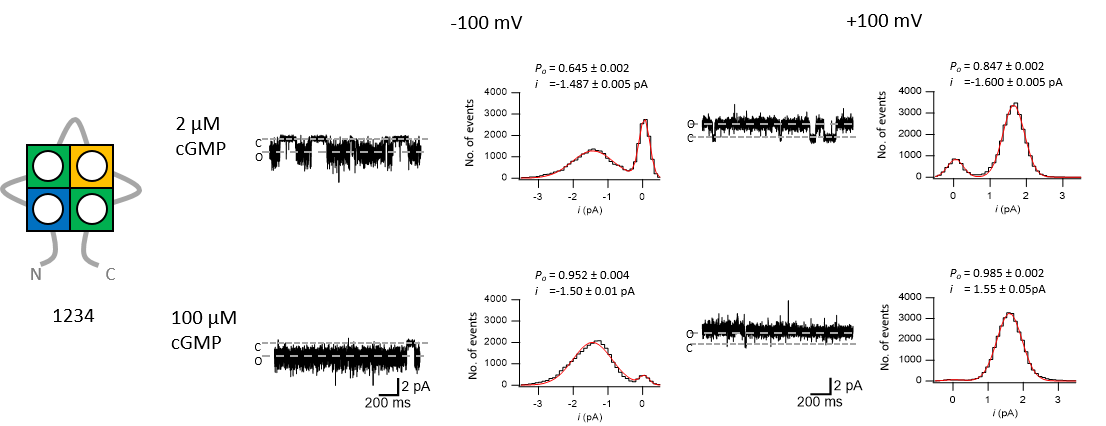
**

**Fig. S8. Single-channel activity in the wt concatamer N-A4-A2-B1b-A2-C (1234).** Data were recorded at either +100 mV or -100 mV at the indicated concentrations of 2 μM or 100 μM cGMP. The recordings were filtered to 5 kHz. The all-point amplitude histograms were fitted with the sum of two normalized Gaussian functions, yielding *P*_o_ and *i*. cGMP controls the open probability, *P*_o_, but not the amplitude of the unitary current, *i*. For display, all data were filtered to 1 kHz. C and O denote the closed and open current level, respectively.
