# Supplementary material for: Subunit promotion energies for channel opening in heterotetrameric olfactory CNG channels
Source: PLoS Comput Biol. 2022 Aug 23;18(8):e1010376. doi: 10.1371/journal.pcbi.1010376 (PMC9512249; doi:10.1371/journal.pcbi.1010376)
Supplement: S9 Fig — (DOCX) [file pcbi.1010376.s009.docx]

**
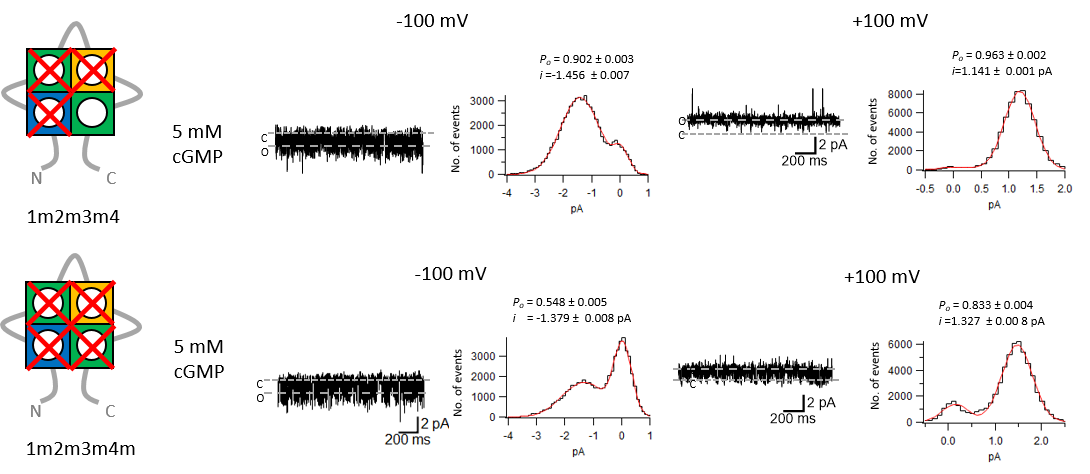
**

**Fig. S9. Single-channel activity in the 1m2m3m4 and 1m2m3m4m concatamer.** The conditions and the type of analysis correspond to Fig. S8.
