# Supplementary material for: Subunit promotion energies for channel opening in heterotetrameric olfactory CNG channels
Source: PLoS Comput Biol. 2022 Aug 23;18(8):e1010376. doi: 10.1371/journal.pcbi.1010376 (PMC9512249; doi:10.1371/journal.pcbi.1010376)
Supplement: S10 Fig — (DOCX) [file pcbi.1010376.s010.docx]

**
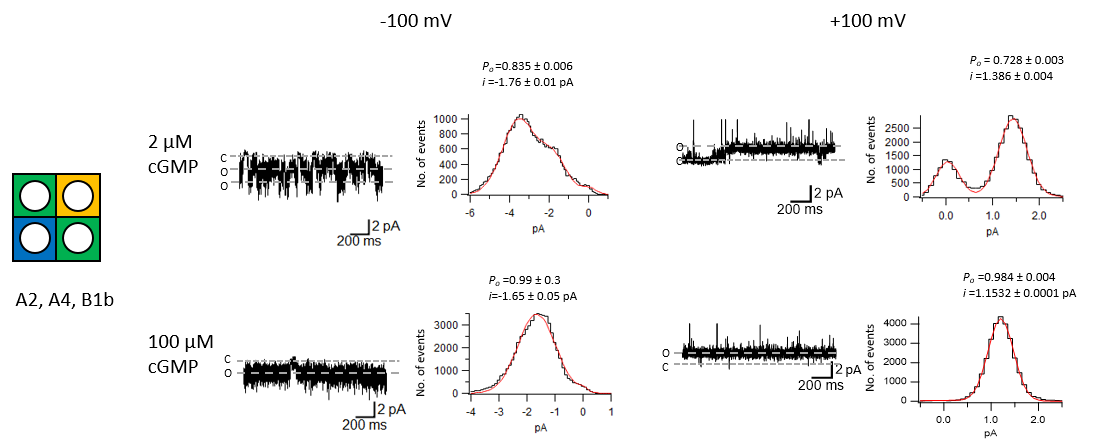
**

**Fig. S10. Single-channel activity in non-concatenated CNGA2:A4:B1b channels.** The corresponding RNA was injected in the ratio 2:1:1. The conditions and the type of analysis correspond to Fig. S8. The patch -100 mV and 2 μM cGMP (upper left) contained two channels.
