# Supplementary material for: Subunit promotion energies for channel opening in heterotetrameric olfactory CNG channels
Source: PLoS Comput Biol. 2022 Aug 23;18(8):e1010376. doi: 10.1371/journal.pcbi.1010376 (PMC9512249; doi:10.1371/journal.pcbi.1010376)
Supplement: S1 Table — (DOCX) [file pcbi.1010376.s011.docx]

**
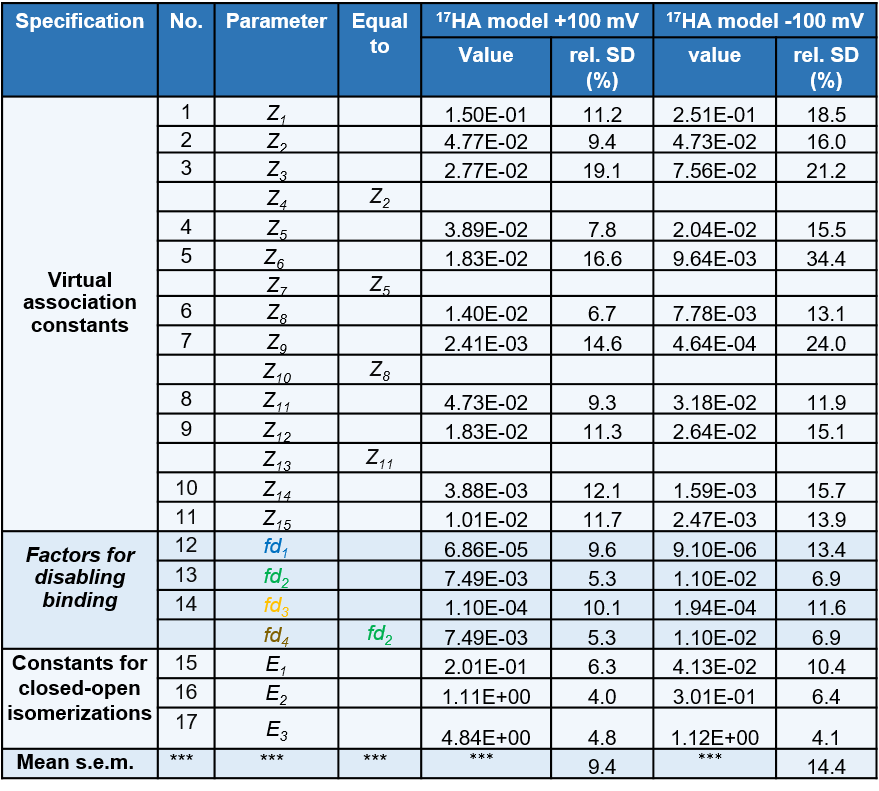
**

**Table S1. Fit parameters of the global fit with the ^17^HA model.** The equilibrium constants correspond to the schemes shown in Figure 1D and Figures S2-S5. The dimensions of *Z_1_-Z_4_*, *Z_5_-Z_10_*, *Z_11_-Z_14_*, and *Z_15_* are μM^-1^, μM^-2^, μM^-3^, and μM^-4^, respectively. *E_1_-E_3_* and *fd*_1_-*fd*_3_ are dimensionless.
