# Supplementary material for: Subunit promotion energies for channel opening in heterotetrameric olfactory CNG channels
Source: PLoS Comput Biol. 2022 Aug 23;18(8):e1010376. doi: 10.1371/journal.pcbi.1010376 (PMC9512249; doi:10.1371/journal.pcbi.1010376)
Supplement: S2 Table — (DOCX) [file pcbi.1010376.s012.docx]

**
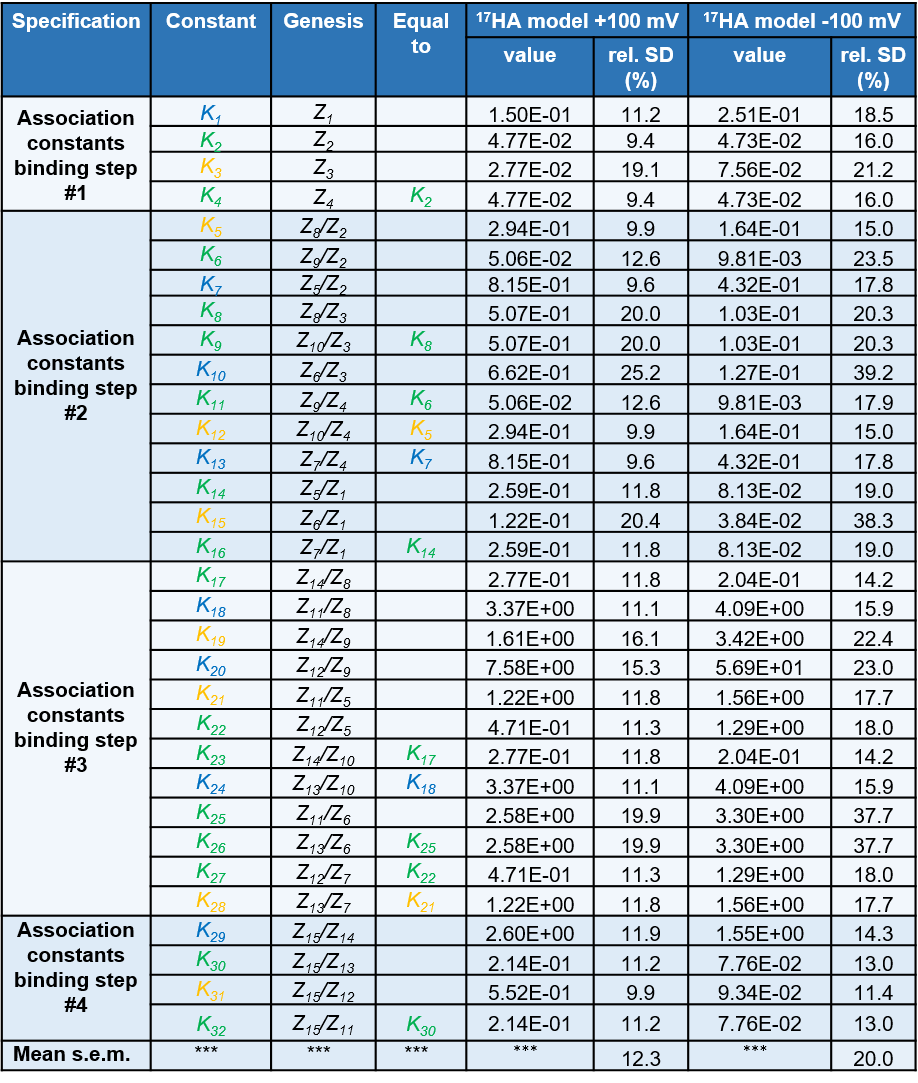
**

**Table S2. Equilibrium constants derived from the global fit with the ^17^HA model.** The constants *K_x_* were obtained from the *Z_x_* in Table S1 as indicated. *K_x_* values are given in μM^-1^.
