# Supplementary material for: Subunit promotion energies for channel opening in heterotetrameric olfactory CNG channels
Source: PLoS Comput Biol. 2022 Aug 23;18(8):e1010376. doi: 10.1371/journal.pcbi.1010376 (PMC9512249; doi:10.1371/journal.pcbi.1010376)
Supplement: S6 Table — (DOCX) [file pcbi.1010376.s016.docx]

**
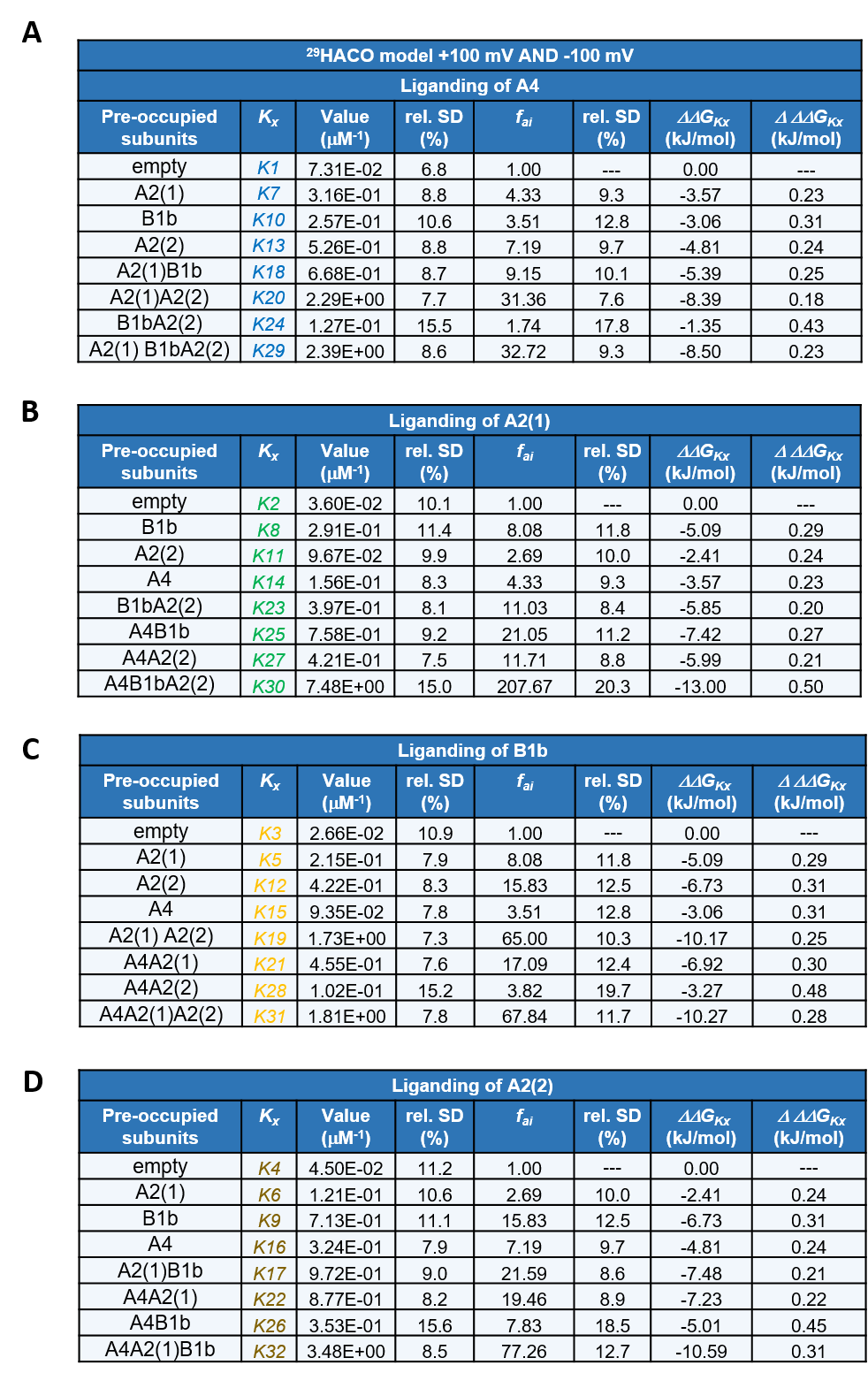
**

**Table S6. Influence of pre-occupation on ligand binding for global fit with the ^29^HACO model at +100 mV AND -100 mV.** (**A-D**) The tables contain at the left side the constants *K_x_* for occupying the subunits A4 (a), A2(1) (b), B1b(c), and A2(2) (d), respectively, at different pre-occupation (bottom). The columns *f_ai_* specify how many times the affinity of a subunit is increased at a given pre-occupation of the other subunits. Both types of data are plotted in Fig. 3A-D. The right column shows the respective free energies obtained by equation (15). The respective errors are indicated.
