# Supplementary material for: Subunit promotion energies for channel opening in heterotetrameric olfactory CNG channels
Source: PLoS Comput Biol. 2022 Aug 23;18(8):e1010376. doi: 10.1371/journal.pcbi.1010376 (PMC9512249; doi:10.1371/journal.pcbi.1010376)
Supplement: S7 Table — (DOCX) [file pcbi.1010376.s017.docx]

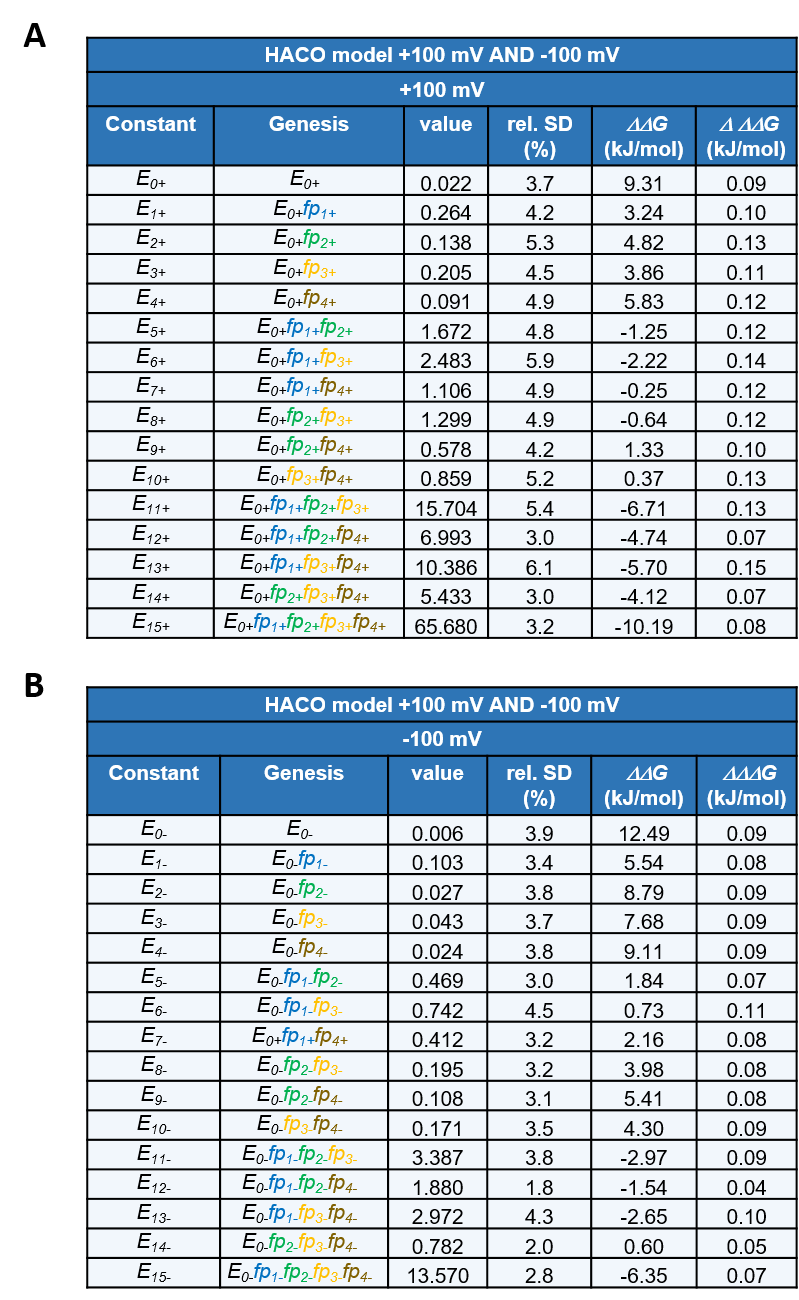


**Table S7. Closed-open isomerization constants given by the global fit with the ^29^HACO model at +100 mV AND -100 mV.** Left columns: Constants *E_0+_*-*E_15+_* and *E_0-_*-*E_15-_* and their errors. The constants are dimensionless. Right columns: Gibbs free energies and their errors. (**A**) +100 mV. (**B**) -100 mV.
