# Supplementary material for: Subunit promotion energies for channel opening in heterotetrameric olfactory CNG channels
Source: PLoS Comput Biol. 2022 Aug 23;18(8):e1010376. doi: 10.1371/journal.pcbi.1010376 (PMC9512249; doi:10.1371/journal.pcbi.1010376)
Supplement: S8 Table — (DOCX) [file pcbi.1010376.s018.docx]

**
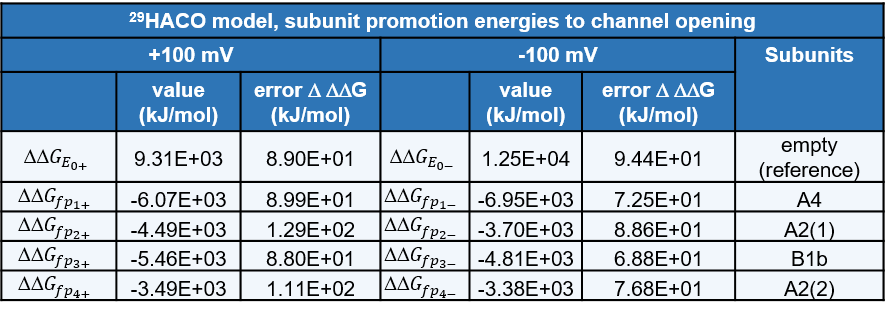
**

**Table S8. Subunit promotion energies for channel opening given by the global fit with the ^29^HACO model at +100 mV AND -100 mV.** The values of *E_0+_*, *fp*_1+_-*fp*_4+_, *E_0-_*, and *fp*_1-_-*fp*_4-_ are dimensionless. The free energies, obtained by equation (16), are indicated.
