# Supplementary material for: Subunit promotion energies for channel opening in heterotetrameric olfactory CNG channels
Source: PLoS Comput Biol. 2022 Aug 23;18(8):e1010376. doi: 10.1371/journal.pcbi.1010376 (PMC9512249; doi:10.1371/journal.pcbi.1010376)
Supplement: S9 Table — (DOCX) [file pcbi.1010376.s019.docx]

**
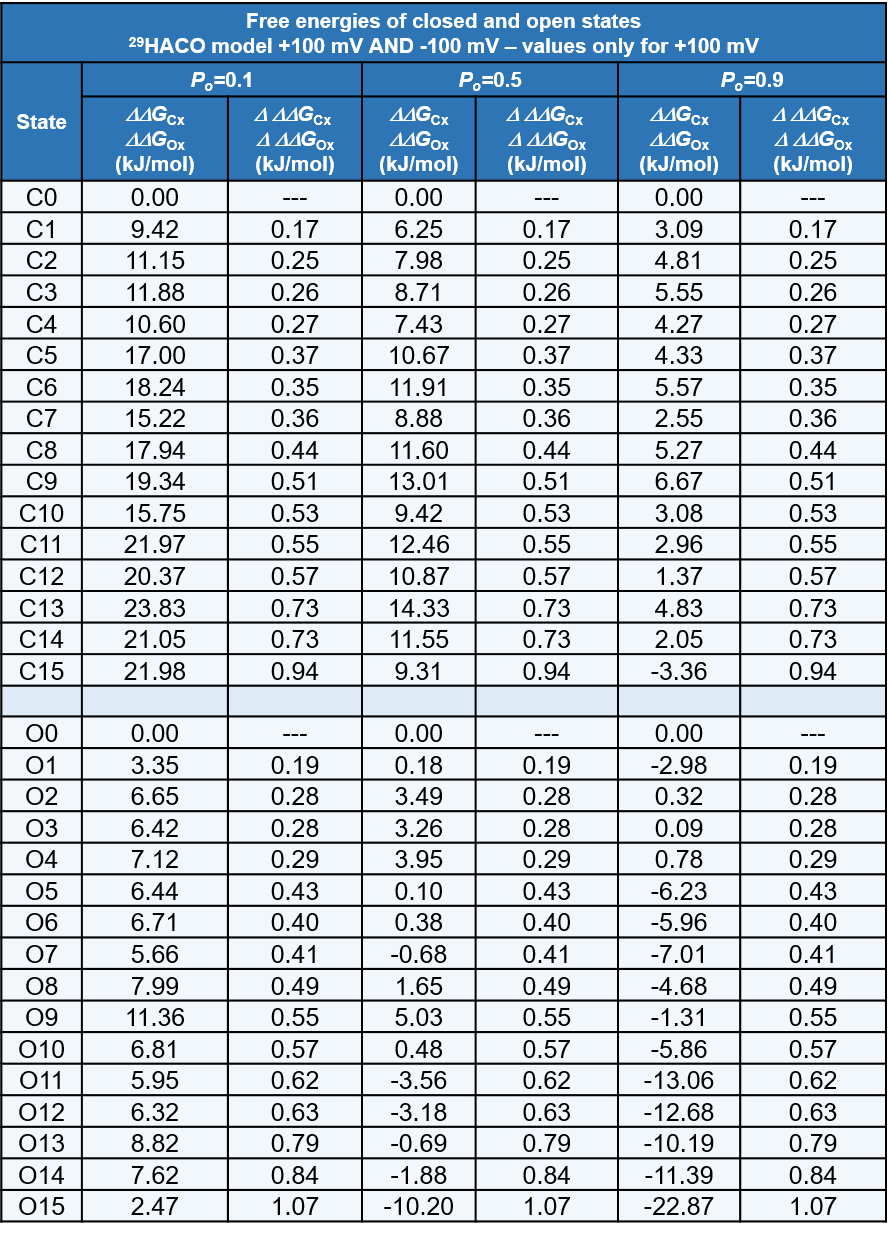
**

**Table S9. Free energies of closed and open states obtained by globally fitting the ^29^HACO model +100 mV AND -100 mV.** The energies of the closed states C1-C15 as well as O1-O15 were related to the reference states C0 and O0, respectively. The values are also plotted in Figure 5 for three ligand concentrations. The energies were computed by equations (17) and (19).
