# Supplementary material for: Subunit promotion energies for channel opening in heterotetrameric olfactory CNG channels
Source: PLoS Comput Biol. 2022 Aug 23;18(8):e1010376. doi: 10.1371/journal.pcbi.1010376 (PMC9512249; doi:10.1371/journal.pcbi.1010376)
Supplement: S10 Table — (DOCX) [file pcbi.1010376.s020.docx]

**
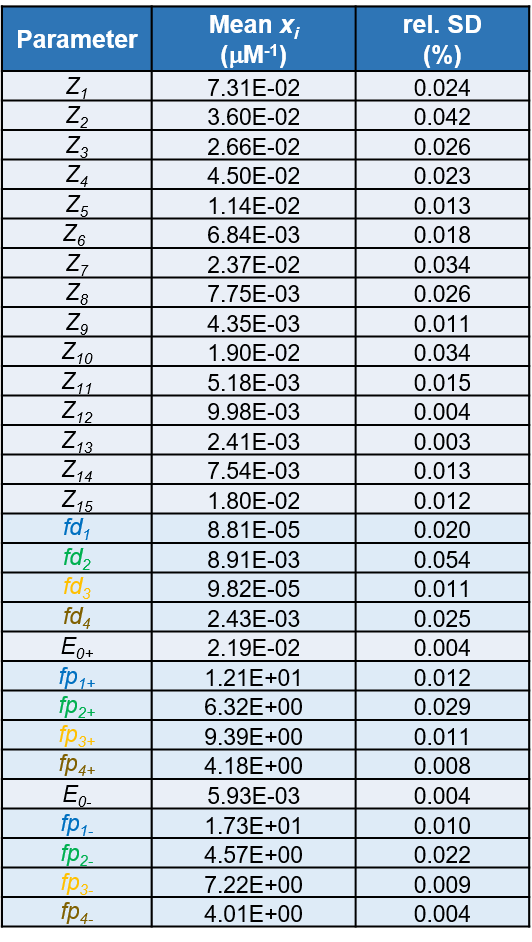
**

**Table S10. Parameters determined with stochastically varied SU start vectors.** The values of the 29 parameters are the means and their standard deviations obtained from 114 successful fits obtained by using SU start vectors between 10^-6^ and 10^0^. First, all fits with negative parameters and subsequently also fits with inconsistent and large minima were discarded (see Methods and Fig. 7 A,B).
