# Supplementary material for: Subunit promotion energies for channel opening in heterotetrameric olfactory CNG channels
Source: PLoS Comput Biol. 2022 Aug 23;18(8):e1010376. doi: 10.1371/journal.pcbi.1010376 (PMC9512249; doi:10.1371/journal.pcbi.1010376)
Supplement: S11 Table — (DOCX) [file pcbi.1010376.s021.docx]

**
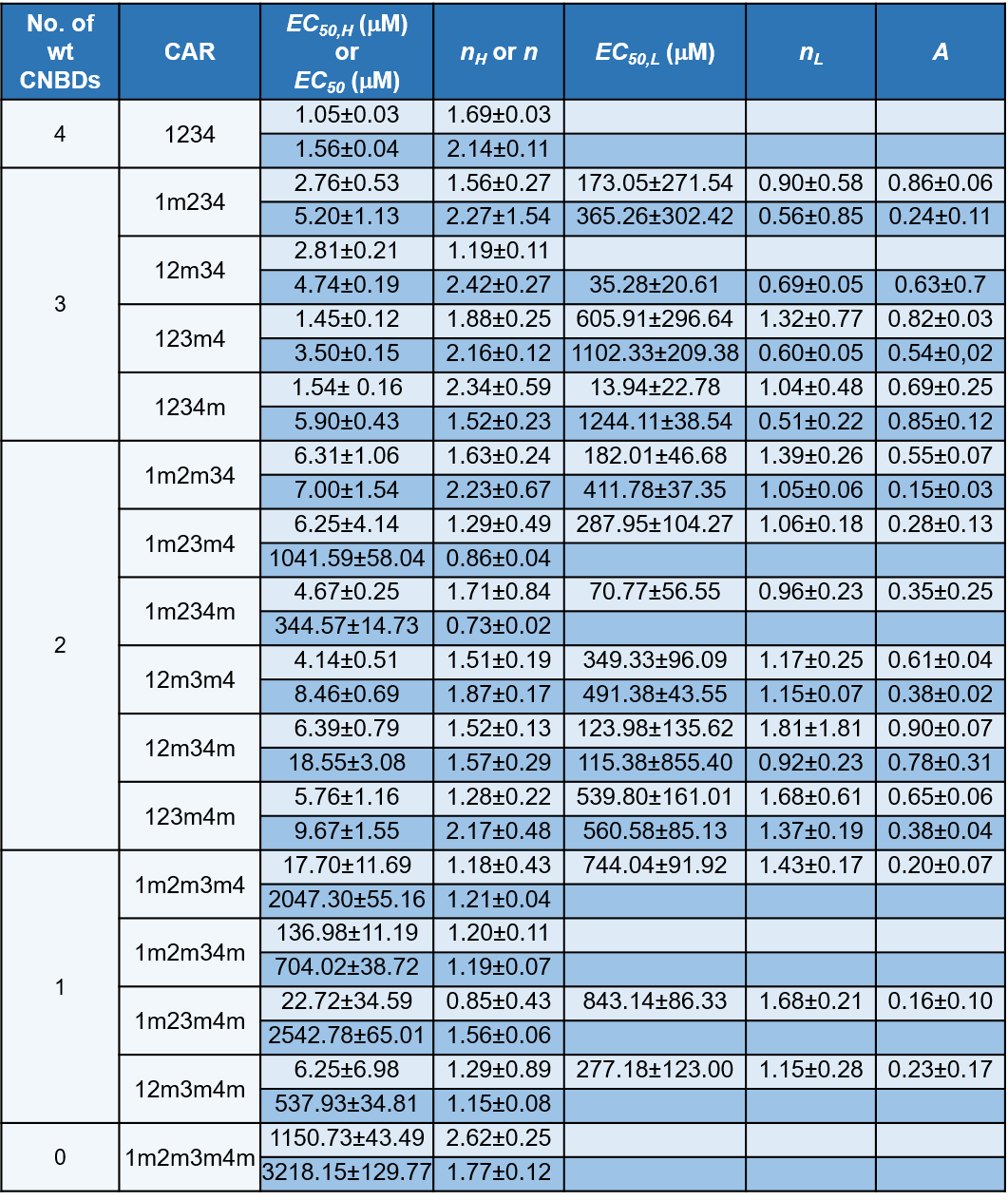
**

**Table S11. Hill parameters for the 32 CARs at +100 mV and -100 mV.** For all 16 concatamers the data points of the CAR at +100 mV and the CAR at -100 mV (see Fig. 2a) were fitted with either a single Hill function (equation 13), yielding half maximum activation, *EC*_50_, and the Hill coefficient, *n*, or the sum of two Hill functions (equation 14), yielding half maximum activation, *EC*_50,H_ and *EC*_50,L_, and the Hill coefficient, *n*_H_ and *n*_L_, for the high-affinity and low-affinity components, respectively. *A* denotes the portion of the high-affinity component. The parameters at +100 and -100 mV are indicated with light and dark blue background, respectively. In total, 124 parameters were required to fit all 32 CARs separately.
