# Supplementary material for: Subunit promotion energies for channel opening in heterotetrameric olfactory CNG channels
Source: PLoS Comput Biol. 2022 Aug 23;18(8):e1010376. doi: 10.1371/journal.pcbi.1010376 (PMC9512249; doi:10.1371/journal.pcbi.1010376)
Supplement: Attachment — Submitted filename: Answers to the Reviewers Rev1_KB3.docx [file pcbi.1010376.s022.docx]

CC: "Jana Schirmeyer" [jana.schirmeyer@web.de](mailto:jana.schirmeyer@web.de), "Thomas Eick" [thomas.eick@med.uni-jena.de](mailto:thomas.eick@med.uni-jena.de), "Eckhard Schulz" [e.schulz@hs-sm.de](mailto:e.schulz@hs-sm.de), "Sabine Hummert" [sabine.hummert@med.uni-jena.de](mailto:sabine.hummert@med.uni-jena.de), "Christian Sattler" [christian.sattler@med.uni-jena.de](mailto:christian.sattler@med.uni-jena.de), "Ralf Schmauder" [ralf.schmauder@med.uni-jena.de](mailto:ralf.schmauder@med.uni-jena.de)

Dear Dr. Hospital,

Thank you very much for submitting your manuscript "Relating energetic opening momenta to ligand binding in individual subunits of heteromeric olfactory CNG channels" for consideration at PLOS Computational Biology.

As with all papers reviewed by the journal, your manuscript was reviewed by members of the editorial board and by several independent reviewers. In light of the reviews (below this email), we would like to invite the resubmission of a significantly-revised version that takes into account the reviewers' comments.

We cannot make any decision about publication until we have seen the revised manuscript and your response to the reviewers' comments. Your revised manuscript is also likely to be sent to reviewers for further evaluation.

When you are ready to resubmit, please upload the following:

[1] A letter containing a detailed list of your responses to the review comments and a description of the changes you have made in the manuscript. Please note while forming your response, if your article is accepted, you may have the opportunity to make the peer review history publicly available. The record will include editor decision letters (with reviews) and your responses to reviewer comments. If eligible, we will contact you to opt in or out.

[2] Two versions of the revised manuscript: one with either highlights or tracked changes denoting where the text has been changed; the other a clean version (uploaded as the manuscript file).

Important additional instructions are given below your reviewer comments.

Please prepare and submit your revised manuscript within 60 days. If you anticipate any delay, please let us know the expected resubmission date by replying to this email. Please note that revised manuscripts received after the 60-day due date may require evaluation and peer review similar to newly submitted manuscripts.

Thank you again for your submission. We hope that our editorial process has been constructive so far, and we welcome your feedback at any time. Please don't hesitate to contact us if you have any questions or comments.

Sincerely,

Alexander MacKerell
Associate Editor
PLOS Computational Biology

Jason Haugh
Deputy Editor
PLOS Computational Biology

Reviewer's Responses to Questions

**Comments to the Authors:
Please note here if the review is uploaded as an attachment.**

We like to thank Reviewer #1 for the criticism which helped us to improve our manuscript in various respects. We appreciate in particular the criticism of the term ‘momentum’. This criticism led us correct our terminology.

Reviewer #1: The MS “Relating energetic opening momenta to ligand binding in individual subunits of heteromeric olfactory CNG channels” by Jana Schirmeyer et al deals with an important topics in the biophysics of olfactory CNG channels. These channels are heteromeric and are formed by two alpha CNGA2 subunits (A2) one CNGA4 (A4) and one CNGB1b. In the present MS, the authors describe the results they have obtained by using the concatemers N-A4-A2-B1b-A2-C concatemers where they have selectively replaced an Arginine in the CN binding site into a Glutamate so that cAMP cannot bind any more.
I like the scope and the experiments here described, but the MS in its present form is rather obscure and even though I am familiar with the topic I was not able to understand it properly. Therefore I suggest to have a mayor revision and here below are my suggestions:

1 - In the introduction it is useful and almost necessary to explain the motivations for building such model and which new insights and/or a better understanding are expected.

We included at the end of the introduction a respective paragraph. It reads

*To better understand the molecular machinery underlying the activation gating in olfactory CNG channels by our strategy of concatenation, subunit disabling, and global fitting is highly attractive because this might answer the question why these three types of homologue subunits are so different in their function. In particular, it is not clear which parts of the subunits cause these significant functional differences and which parts do interact to generate the cooperative activation and which parts do not. Furthermore, continuative analyses of our unusually consistent data set of 16 concatamers with systematically disabled subunits provides a chance to fathom in a more general way to what degree of detail such constructs can be exploited to functionally analyze heterooligomeric channels with per se silent subunits.*

2 - there must be a paragraph clearly stating the assumptions on which the model/analysis is based

We followed the suggestion of the reviewer and better outlined the major assumptions for our fit.

At the beginning of the results part it reads now

*In contrast to our previous report [1], we assumed here that the E_x_ can be disassembled to elementary subunit components. An elementary subunit component is specified by the promotion factor for opening, fp_n_ (n=1…4), shifting the closed-open isomerization of the empty channel, specified by the constant E_0_, more to the open state,*

$E_{x}=E_{0} \prod_{1}^{4} {fp}_{n}$	.	(1)

*For empty subunits, the* *fp_n_ are unity, whereas for occupied subunits the corresponding fp_n_ have values >1 and are specific.* *Hence, the 16 E_x_ are generated by 16 combinational products of the 5 parameters E_0_, fp_1_…fp_4_.*

Furthermore, we tried to clarify the introduction of the first part in the section ‘Subunit promotion energies to channel opening’ (former headline ‘Energetics of subunit opening momenta’). It reads now

*To ease the considerations, all 16 E_x_ were translated to Gibbs free energies (Table S7 A,B) according to*

${\Delta\Delta G}_{Ex} =-RT\ln(E_{0}\prod_{1}^{4} {fp}_{n})=-RT\ln(E_{0})-RT\sum_{1}^{4} ln\left( {fp}_{n} \right)$*.	(2)*

*R and T are the molar gas constant and the temperature in K. Thus, the four subunit-specific promotion factors fp_n_ are translated to free energies,* ${\Delta\Delta G}_{{fp}_{n}}$*, termed ‘subunit promotion energies on channel opening’ in the following. They now add in a combinational way to* ${\Delta\Delta G}_{E_{0}}$*. For the two voltages of +100 mV and -100 mV, this results in two sets of 16* ${\Delta\Delta G}_{Ex}$*built by* ${\Delta\Delta G}_{E_{0+}}$*and* ${\Delta\Delta G}_{{fp}_{n+}}$ *as well as* ${\Delta\Delta G}_{E_{0-}}$*and* ${\Delta\Delta G}_{{fp}_{n-}}$*, respectively.*

3 - In the discussion it is necessary to evaluate the conclusions gained by such modelling/computation
We followed the suggestion of the reviewer and re-wrote and extended part of the discussion to point out to what extent our conclusions depend on the model assumptions. The text reads now:

*As in any case of interpretation of data by a model, also our interpretations depend on the specificities of the used ^29^HACO model. We like to state again that the ^29^HACO model seems to us highly plausible for a heterotetrameric channel with four binding sites despite its considerable complexity. The key assumptions of this model are:*

(1) *We assigned the moderate effects of voltage exclusively to the closed-open isomerizations (E_x_) of the channel at the different combinations of liganding. This assumption is highly plausible because the closed-open isomerizations proceed in the channel core subjected to the transmembrane electric field but not in the binding reactions (K_x_) proceeding in the CNBDs located in the cytosol, and thus outside the transmembrane electric field.*
(2) *For our HA models, underlying the complex ^29^HACO model, we assumed that the four subunits in the channel exert only a binary closed-open action in a highly cooperative way, leading to a single opening step in our models. This assumption was supported by the observation, that the ligand concentration controls solely the open probability, P_o_, but not the single-channel conductance (Figs. S8-S10). We therefore did not consider models of the Koshland-Nemethy-Filmer (KNF) type [2, 3].*
(3) *Our approach assumed that* ${\Delta\Delta G}_{{fp}_{n}}$*of each occupied subunit is independent of the occupancy of the other subunits. Thus, we reduced the complex interaction of the subunits in the closed-open isomerizations into four independent functional modules. This assumption is a priori not without alternative. The success of our fit, however, suggested to us that this assumption is reasonable.*
(4) *Modular functionality within the protein was also assumed for the disabling factors fd_x_, i.e. they were taken as independent of the occupation of the other subunits [1]. Again, the success of our fit suggested to us that this assumption is reasonable.*

*Under these model assumptions, the phenomenon of subunit cooperativity would be confined to the level of the CNBDs. This interpretation gets nice support from recent computational studies on the allosteric signaling within the CNBD and the C-linker of structurally related HCN2 channels [4]. The authors applied MD simulations and a rigidity-theory-based approach and identified complex interactions to mediate the cAMP effect. They identified in the CNBD and the C-linker two intersubunit pathways and one intrasubunit pathway. Our analysis leaves open which structures further process the information from the CNBD and the C-linker to the pore but suggests that that this process runs independent in each subunit, i.e. there is no major additional cooperative interaction of the subunits at the level of the channel core.*

*The overall view is the following: Ligand binding to the different subunits of a heterotetrameric olfactory channel appears with different affinity, independent of the state of the other subunits but highly sensitive to mutagenesis (Fig. 6). In contrast, subsequent conformational changes in the tetrameric CNBD modulate the affinity of the CNBDs with high specificity in a cooperative fashion whereas the specific* ${\Delta\Delta G}_{{fp}_{n}}$*of a subunit, controlling the channel pore, is independent again.*

The above three suggestions are - in my view - compulsory and now I list some questions for the authors. Indeed they have the data to answer either in a final and definitive way or at least to provide a substantial better understanding for several important issues :
from the data presented in the MS it seems that some channel opening are observed when all or most of the CN binding sites are knocked off by the mutation R/E. Do the authors have some single channel recordings from these concatened and mutated channels? If so I will be delighted to see these recordings.

It is an important aspect of our whole analysis that mutating a subunit does not mean to knock the binding out. Instead, it means to disable the binding site in the sense that the concentration-activation-relationship is shifted to much higher concentrations. As a consequence, we could also obtain CARs from the concatamers with triple and quadruple mutations and these CARs are also shown in Fig. 2.

To further substantiate this and to follow the suggestion of the reviewer we added examples of single-channel recordings for the triple and quadruple disabled construct 1m2m3m4 and 1m2m3m4m, respectively (new Figs. S9 and S10). Moreover, we now provide reference single-channel recordings from non-concatenated wild-type channels (Fig. S11).

in long single channel recordings from the CNGA1 monomeric channel it is possible to observe occasionally long closures. Have the authors observed anything similar in their concatenated channels? If so is this observation compatible with the assumptions of their modelling?

To our opinion long shut times appear in each type of single-channel recording if a channel falls to a sleeping mode. We observed such events very rarely and do not have evidence that such events appear more often in concatenated channels. Because our CARs were obtained from ensemble currents with hundreds of channels, we assumed that statistically the appearance of such sleeping events is invariant in time and reduces thus the current amplitude at all ligand concentrations proportionally. We therefore did not further consider these events in our modeling.

In the MS there is a number of words, which are awkward with a not obvious meaning such:

The authors refer to Energetics of subunit opening momenta. The word “momenta” in science is the product of the mass times the velocity and I presume that the authors do not intend this.
The words endergonic and exergonic are often used and their meaning must be defined or – better – more conventional words must be used.

We particularly thank the reviewer for this criticism because our wording was indeed misleading. A momentum has the dimension kgm/s and is not an energy. What we mean is indeed a free energy. We therefore changed the wording in the whole manuscript and eliminated the term ‘momentum’. We specify our energies now as ‘subunit promotion energies for channel opening - ${\Delta\Delta G}_{{fp}_{n}}$’. We changed also the title of the manuscript accordingly. In particular, we also elaborated that the subunit promotion factors *fp_n_* are translated into ‘subunit promotion energies for channel opening, ${\Delta\Delta G}_{{fp}_{n}}$’ by modifying equation 2. Now the terms endergonic and exergonic have their correct meaning.

In the legend of Fig.1 it is stated:
“ Scheme with two closed and two open states (C0, C1, O0, O1) for a theoretical minimum ligand-gated receptor containing for the closed channel one binding step (K1) and two allosteric opening steps “
All this is hard to understand and very confusing and leaves the impression of a rather sloppy writing/editing, as if the MS is a preliminary draft and not a finished and well-polished MS

We revised the description to increase clarity. It reads now:

*Scheme with two closed and two open states (C0, C1, O0, O1) for a theoretical minimum ligand-gated channel. There is one ligand binding step from closed state C0 to closed state C1, specified by the equilibrium association constant K_1_. From each closed state an opening step is possible that is specified by the respective closed-open isomerization constant E_0_ and E_1_.*

Reviewer #2: Understanding the complexities of heteromeric ion channel gating has been difficult and many electrophysiological studies to measure heteromeric CNG channel currents have been reported. Here, Schirmeyer and authors refine recently generated models to understand the energetic contributions of individual subunits to gating by ligand binding and voltage. This is an interesting question and a unique way to look at allostery and cooperatively in these channels.

We like to thank Reviewer #2 for the helpful criticism which helped us to improve the manuscript.

The same concatemers from Schirmeyer et al. 2021 and the resulting patch clamp data recorded at +100 mV were used for both Benndorf et al. 2022 JGP and this current manuscript.

For the computational model in this manuscript, the authors add patch clamp data for the same constructs recorded at -100 mV. They fit 32 equilibrium association constants and 16 equilibrium isomerization constants in contrast to the 32 equilibrium association constants and 4 equilibrium isomerization constants (Benndorf et al. 2022 JGP). Most of the results expound upon the additional and new fit constants or derivations thereof (free energies). Some of these fit constants are similar while others are more variable between the previous HA (+100 mV) and HACO models (+100 mV and -100 mV).

We like to note that the JGP paper Benndorf et al. is a pure theoretical study to show why such an extensive global fit could indeed produce such an amount of determined parameters at all. Initially, we were totally surprised by ourselves by this fact. Indeed, this JGP paper does not provide new information on the equilibrium constants as determined in the previous PNAS paper Schirmeyer et al.

Compared to the paper Schirmeyer et al., the present paper includes actually new data at another voltage and uses also another model (^29^HACO model) by exploiting a mild voltage dependence. To our opinion, it is not surprising that parameters in the new model moderately deviate from those obtained with the former model.

Notably, with the ^29^HACO model we did not fit 16 independent parameters for the 16 closed-open isomerizations but chose a combinational approach using only 5 parameters per voltage. The parameters are now termed ‘subunit promotion factors’ or after translation into free energies, ‘subunit promotion energies for channel opening’.

The term ‘energetic momentum’ has been eliminated due to the criticism of Reviewer #1. This combinational approach was just an assumption of the ^29^HACO model but, again to our surprise, it turned out that the determinateness of these parameters was remarkably high.

Major point

Improvement in fitting are less than 5% relative standard deviations (Figure S6) or approximately equivalent to the error of estimated constants (Table 1), which I believe reduces the enthusiasm for the actual enhancement of the HACO model (+100 mV and -100 mV) over the HA model (+100 mV). Could the authors comment on this?

We do not see that the major value of the fit with the ^29^HACO model is to compare relative standard deviations between different model. In our eyes the major advancement associated with the ^29^HACO model is that the assumption of additive ‘subunit promotion energies for channel opening’ seemed is reasonable. Otherwise, we either would not have obtained reasonable fits or the determinateness of the parameters would not have been in a reasonable range. Moreover, the global fit with the ^29^HACO model at +100 and -100 mV has 12 (!) free parameters more than the previous fit of the data at +100 mV only with the ^17^HA model and the mean SDs are even smaller. We take this as indicator that the adequacy of our ^29^HACO model is high. Finally, as a side finding we obtained information about the voltage dependence of the closed-open isomerizations of the different subunits when comparing corresponding *fp_n-_* and *fp_n+_* values (Table 1).

Minor points

Why are equivalent KCl concentrations in the bath and pipette for the patch clamp experiments?

In fact, symmetrical K^+^ is our standard condition for recording currents of CNG channels in such biophysical approaches. Symmetrical conditions ease interpretation when comparing current amplitudes at different voltages because the driving force is linear in contrast to asymmetric conditions where one has to take into account nonlinear relationships according to the GHK equation. Alternatively, one could have used also symmetric Na^+^.

Show single channel activity of natural CNGs at -100 mV to complement Figure S8. It would also be nice to see representative comparisons for mutants between natural and concatemer channels at the different Po's described at both -100 mV and +100 mV.

We included now in the new Figs. S9 and S10 single-channel recordings from triple mutated (1m2m3m4) and quadruple mutated (1m2m3m4m) concatamers and, for comparison, also from non-concatenated wt channels (Fig. S11). These figures demonstrate that the single-channel conductance is neither affected by concatenation nor by the disabling mutations.

We included a respective sentence in the Methods section:

*Example single-channel recordings of wt concatamers, triple-mutated concatamers, quadruple-mutated concatamers as well as non-concatenated channels are shown in Figs. S8 to S11, respectively.*

While these concatemers were used in previous publications to generate models, are the authors concerned with the differences in kinetics between the natural CNG channel and the concatemer and what this could mean for their conclusions (Figure 2A and 2B)?

When taking into consideration the different time bars in Fig. 2A and B the, the kinetics are indeed closely similar. We did not notice relevant differences and therefore did not systematically analyze the kinetics. Even if there would be minor differences, our type of analysis of equilibrium data would certainly not be affected.

Can the authors speculate why the constants are so different between +100 mV and -100 mV?

In fact CNG channels are classified as voltage-independent channels though they belong to the superfamily of VGICs. It seems to be clear now that the VSD does not relevantly couple to the pore domain. Nevertheless, closer inspection of the current traces reveals a weak voltage dependence and it can be demonstrated directly by the time-dependent components in the currents when switching from -100 mV to -100 mV (Fig. 2A,B). For CNGA1 channels we determined previously an apparent gating charge of 0.23 [5] which is indeed low. We estimate that similar values could be determined for both CNGA2 and CNGA2:A4:B1b channels.

To speculate about the origin of the voltage dependence, it is of course possible that the VSD still does exert a minor effect on the pore. Alternatively, it is also possible that such a minor voltage dependence is caused by the pore domain itself because it also contains charged amino acids. Experimentally, one might figure this out by neutralizing some charges either in the VSD or the pore domain.

**Have the authors made all data and (if applicable) computational code underlying the findings in their manuscript fully available?**
The [PLOS Data policy](https://journals.plos.org/ploscompbiol/s/materials-and-software-sharing) requires authors to make all data and code underlying the findings described in their manuscript fully available without restriction, with rare exception (please refer to the Data Availability Statement in the manuscript PDF file). The data and code should be provided as part of the manuscript or its supporting information, or deposited to a public repository. For example, in addition to summary statistics, the data points behind means, medians and variance measures should be available. If there are restrictions on publicly sharing data or code —e.g. participant privacy or use of data from a third party—those must be specified.

Reviewer #1: **No:** I have not seen neither the code nor the original data, but they could be somewhere and I did not notice

Reviewer #2: Yes

All data and codes underlying the findings are available at <https://osf.io/zcuyv>.

PLOS authors have the option to publish the peer review history of their article ([what does this mean?](https://journals.plos.org/ploscompbiol/s/editorial-and-peer-review-process#loc-peer-review-history)). If published, this will include your full peer review and any attached files.


If you choose “no”, your identity will remain anonymous but your review may still be made public.


**Do you want your identity to be public for this peer review?** For information about this choice, including consent withdrawal, please see our [Privacy Policy](https://www.plos.org/privacy-policy).

Reviewer #1: **Yes:** Vincent Torre

Reviewer #2: No

 Figure Files:

While revising your submission, please upload your figure files to the Preflight Analysis and Conversion Engine (PACE) digital diagnostic tool, [https://pacev2.apexcovantage.com](https://pacev2.apexcovantage.com/). PACE helps ensure that figures meet PLOS requirements. To use PACE, you must first register as a user. Then, login and navigate to the UPLOAD tab, where you will find detailed instructions on how to use the tool. If you encounter any issues or have any questions when using PACE, please email us at [figures@plos.org](mailto:figures@plos.org).

We subjected all figures to the PACE and the PLOS requirements are met.

Data Requirements:

Please note that, as a condition of publication, PLOS' data policy requires that you make available all data used to draw the conclusions outlined in your manuscript. Data must be deposited in an appropriate repository, included within the body of the manuscript, or uploaded as supporting information. This includes all numerical values that were used to generate graphs, histograms etc.. For an example in PLOS Biology see here: <http://www.plosbiology.org/article/info%3Adoi%2F10.1371%2Fjournal.pbio.1001908#s5>.

We declare at the end of the main manuscript:

## Data Availability

Custom Matlab code and data used for the global fit have been deposited in Open Science Framework (<https://osf.io/zcuyv>). All other data are included in the manuscript and/or Supplementary Information.

Reproducibility:

To enhance the reproducibility of your results, we recommend that you deposit your laboratory protocols in protocols.io, where a protocol can be assigned its own identifier (DOI) such that it can be cited independently in the future. Additionally, PLOS ONE offers an option to publish peer-reviewed clinical study protocols. Read more information on sharing protocols at <https://plos.org/protocols?utm_medium=editorial-email&utm_source=authorletters&utm_campaign=protocols>

*In compliance with data protection regulations, you may request that we remove your personal registration details at any time.* [*(Remove my information/details)*](https://www.editorialmanager.com/pcompbiol/login.asp?a=r)*. Please contact the publication office if you have any questions.*

**References for the answers**

1.	Schirmeyer J, Hummert S, Eick T, Schulz E, Schwabe T, Ehrlich G, et al. Thermodynamic profile of mutual subunit control in a heteromeric receptor. Proc Natl Acad Sci U S A. 2021;118(30). Epub 2021/07/25. doi: 10.1073/pnas.2100469118. PubMed PMID: 34301910; PubMed Central PMCID: PMCPMC8325370.

2.	Koshland DE, Jr., Nemethy G, Filmer D. Comparison of experimental binding data and theoretical models in proteins containing subunits. Biochemistry. 1966;5(1):365-85. Epub 1966/01/01. PubMed PMID: 5938952.

3.	Levitzki A, Koshland DE, Jr. Negative cooperativity in regulatory enzymes. Proc Natl Acad Sci U S A. 1969;62(4):1121-8. Epub 1969/04/01. doi: 10.1073/pnas.62.4.1121. PubMed PMID: 5256410; PubMed Central PMCID: PMCPMC223623.

4.	Pfleger C, Kusch J, Kondapuram M, Schwabe T, Sattler C, Benndorf K, et al. Allosteric signaling in C-linker and cyclic nucleotide-binding domain of HCN2 channels. Biophys J. 2021;120(5):950-63. Epub 2021/01/31. doi: 10.1016/j.bpj.2021.01.017. PubMed PMID: 33515603; PubMed Central PMCID: PMCPMC8008325.

5.	Benndorf K, Koopmann R, Eismann E, Kaupp UB. Gating by cyclic GMP and voltage in the alpha subunit of the cyclic GMP-gated channel from rod photoreceptors. J Gen Physiol. 1999;114(4):477-90.
